# Online Ethnography for People With Chronic Conditions: Scoping Review

**DOI:** 10.2196/37941

**Published:** 2022-11-29

**Authors:** Yajing Gao, Xuemei Chen, Wei Zhang, Qiuyi Wang, Jing Liu, Lanshou Zhou

**Affiliations:** 1 Nursing School of Naval Medical University Shanghai China

**Keywords:** online ethnography, chronic condition, scoping review, review, ethnography, online, research, online users

## Abstract

**Background:**

Online ethnography has been making a unique contribution to people with chronic conditions as a complement to offline ethnography. It can also be used to study the complexities and contingencies of people with chronic conditions in the context of the internet. Therefore, there is a need to synthesize existing knowledge on research activities concerning online ethnography for people with chronic conditions.

**Objective:**

This scoping review aimed to profile the existing evidence on the application of online ethnography for people with chronic conditions, focusing on the characteristics, contributions, and implementation process. This will provide recommendations for the future use of online ethnography.

**Methods:**

We followed the scoping review methodologies developed by Arksey and O’ Malley and the Joanna Briggs Institute. A comprehensive search was conducted on the PubMed, CINAHL, Embase, Scopus, and PsycInfo databases using preselected keywords. The search was limited to documents written in English and published between January 1, 2000, and February 1, 2022. After removal of duplicates, articles were screened by 2 independent reviewers reading the title, abstract, and full text. One reviewer extracted data, which were descriptively analyzed to map the existing knowledge.

**Results:**

After 2836 titles and abstracts and 51 full texts were screened, 27 publications were included in the analysis, published between 2009 and 2022. Most studies were from the United States (11/27, 40.7%), and most articles collected data from online forums (10/27, 37.0%). Moreover, the most commonly used type of researcher involvement was passive analysis (24/27, 88.9%), and 18.5% (5/27) of the topics concerned people with mental illness. Notably, the majority of articles did not report the immersion process in detail (17/25, 63.0%). Ethical issues were mentioned in 88.9% (24/27) of the included articles.

**Conclusions:**

We analyzed the current literature across fields and found that online ethnography can be exploited to explore the deeper experience of people with chronic conditions that are difficult to investigate using traditional ethnography. We found that there was diversity in researcher involvement, immersion process, data collection, and data analysis. However, most studies reported the insufficient immersion into the online environment. Researchers should determine the research approaches and data resources in order to complete culture immersion before researching. We also found that there was no uniform standard for ethical issues. Therefore, we recommend that researchers collect public and private data, obtain informed consent, and preserve the privacy and confidentiality of online users with chronic conditions. The findings can provide a practical reference for the use of online health care in studying chronic conditions.

## Introduction

There is an increasing number of patients with chronic conditions worldwide. The use of digital technology for these patients has become common due to advances in technology. People with chronic conditions include patients experiencing chronic physical pain, mental conditions, continuing conditions, chronic symptoms, or alcohol and substance abuse that normally last more than a year [1,2]. Among the chronic conditions, noncommunicable diseases alone accounted for 60.8% of all deaths in 2000, which rose to 73.6% in 2019 globally [3]. Patients with chronic conditions often face varied long-term physical and mental challenges which require long-term professional and nonprofessional care including health information, psychological care, symptom management, and social support [4,5]. Fortunately, the development and extensive use of digital technology has provided a potential avenue to meet the diverse needs of patients with chronic diseases. Indeed, the World Health Organization (WHO) Global Strategy on Digital Health was adopted in 2020 by the World Health Assembly [6]. For these patients, the most convenient way to take advantage of the available digital technology is to access online health communities, social media, government or charity websites, and live chats, which enables them to share, communicate, and search for tailored health information and implementation of health management [7-9]. One of the most important features of the digital platforms is that users in discussion boards can express themselves anonymously, thus providing them with sufficient privacy protection [7]. Moreover, patients are able to talk freely about particular topics which they would otherwise not mention [10]. In addition, the online platforms can store data about the patients’ perceptions and experiences, the interactions between patients, and the interactions between doctors and patients. Moreover, memes and symbols also provide information through nonverbal interactions [11]. Thus, these features have made the online platforms important approaches in collecting qualitative data of patients with chronic conditions.

With the growth of online health communities and platforms, online ethnography has attracted more attention from health scholars, especial those who are interested in chronic diseases. Ethnography research is a qualitative methodology, which allows health professionals and researchers to study and generate a comprehensive understanding of social interaction, behavior, and perceptions that occur within groups, teams, and communities in the health care setting [12]. With a background in anthropology and sociology, ethnography offers a way to understand a particular cultural context. A researcher conducting this kind of study often spends time watching, listening, and engaging with the study participants before analyzing, interpreting, and theorizing upon the observed phenomena. As ethnographic methodologies have flourished, different frameworks supporting different research purposes, settings, and issues have emerged. These frameworks include institutional ethnography, which is focused ethnography and video-ethnography [13-15]. Online ethnography, also referred to as netnography or internet ethnography, has emerged as a new form, along with the metaverse concept, to yield great advantages in the medical field. Nowadays, a massive pool of medical information can rapidly and anonymously be found online, from which someone’s commentary or experience with a specific health or medical issue can provide new research insight. As a fairly new approach, online ethnography uses ethnography in online spaces and communities to explore holistic and cultural thoughts as well as the feelings and experiences of particular groups [16]. In the past two decades, there has been an evolution from “participant-observational research” to “netnography” [17]. Earlier online ethnography held a distinction between online and offline realities and tended to search individuals’ interactions with online spaces by observing them in offline settings. Whereas, nowadays there is a growing preference to use real-time, participatory netnography [18,19]. Netnography improves accessibility to geographically hard-to-reach populations who prefer anonymity, is more convenient, allows for the ultimate naturalistic inquiry, and has no recall bias [16]. Online ethnography and related methods have been used to research health-related topics, which include exploration of cyber-nursing approaches, discussions on image and performance-enhancing drugs, and examinations of UK general practitioners’ views on health policy changes [20-22]. Therefore, a combination of internet-delivered ethnographic modalities and patient-centered approaches is an exciting area that could be pursued in the management of chronic illness, owing to the widespread adoption of online platforms among the patients, including those with mental illness, epilepsy, or diabetes [23-26].

Although there is a growing number of online ethnography studies for people with chronic conditions, there is a lack of reviews synthesizing their common characteristics. In addition, the field of online ethnography is relatively young and thus lacks structures and guidelines, and so many studies are in the exploratory stage. Furthermore, there is limited data on the research specification and standards within a given type of online context and participant group. Consequently, there might be bias in the development of online ethnography. A review of the network ethnography in the field of chronic conditions can help understand research status, existing problems, and issues requiring attention concerning the new research method. Among the different types of reviews, scoping reviews yield a comprehensive review of a new research area [27]. Therefore, we aimed to conduct a scoping review to explore the research status of online ethnography among people with chronic conditions to yield an in-depth understanding of this new method and provide the basis for further research.

## Methods

### Overview

We employed the scoping review framework by Arksey and O’ Malley [28], which encompasses five stages: (1) identification of initial research questions; (2) identification of relevant studies; (3) study selection; (4) charting the data; and (5) collating, summarizing, and reporting the results.

### Stage 1: Identifying the Research Question

The two main review questions were as follows: (1) What are the characteristics and contribution of online ethnography in people with chronic conditions? (2) How should ethnography be conducted for people with chronic conditions in an online environment?

### Stage 2: Identifying Relevant Studies

Initially, a literature search was manually conducted in PubMed to identify chronic disease fields where online ethnography was mostly used and developed. We searched for the term “chronic disease” to identify relevant papers. However, the retrieved results were not comprehensive because of our consideration of “chronic conditions” instead of “chronic diseases.” Therefore, we decided to choose eligible articles from a wide range of articles and then searched in 5 electronic databases: PubMed, CINAHL, Embase, Scopus, and PsycInfo. The search strategy was as follows: (online* OR internet* OR cyber* OR web* OR digital OR online forum OR virtual community OR Facebook OR Twitter OR Instagram OR blog OR Youtube OR social media OR remote video OR visual) AND (nethnography OR ethnography OR netnography). The search included 2 sets of search terms: “online” or “ethnography.” To capture the evolution of online ethnography in people with chronic conditions over the years, the databases were searched between January 1, 2000, and February 1, 2022. An additional list of 2 relevant articles was manually searched to identify any other potentially relevant articles.

### Stage 3: Study Selection

Here, we adopted the Joanna Briggs Institute’s population-concept-context framework to define our inclusion criteria. We included people with chronic conditions and searched for chronic diseases as a general concept as well as specific diseases including stroke, asthma, chronic obstructive pulmonary disease, cancer, and mental disease. We used any other relevant publications regardless of age, origin, or gender of the studied populations. The concept was use of ethnography while the context was online. We included full texts that reported the following contents: online ethnography being taken as a research purpose, collection of data with online ethnography, and analysis of data with online ethnography. This research included articles that were written in English; published between January 1, 2000, and February 1, 2022; and that evaluated online ethnography in people with chronic conditions. We excluded articles that were not directly related to our research review topic, such as those about dying patients and nursing robots. Gray literature and studies about nonhuman subjects were also excluded. In addition, we excluded articles which did not involve people with chronic conditions and those with no relevant information on online ethnography. A 2-step screening protocol was employed after duplicate removal. First, titles and abstracts were screened to determine the eligibility of each article. Full texts were then screened, and only articles that met the eligibility criteria were included. Two reviewers evaluated the articles using title and abstract analysis for relevance to online ethnography in people with chronic conditions. Full texts were retrieved and independently reviewed by 2 authors (WZ and QW) to confirm study eligibility. Consensus was reached through discussion and, where required, with consultation with a third author (LZ). One author (XC) conducted a supplementary hand search of reviews retrieved from the database search to identify additional studies to be included after consensus with the second author (YG).

### Stage 4: Charting the Data

A data extraction template was developed and independently piloted by 2 authors (YG and JL), which was then refined for the purposes of this review. Data were extracted from the included studies by 1 author (YG) and verified by the second author (XC). Information relating to authors, year, study design, country, target group, type of researcher involvement, data source, methods of immersion, data collection, data analysis, study purpose, results, ethical considerations, and limitations were extracted and tabulated using Microsoft Excel 2019 software. Moreover, the type of research was divided according to Keim-Malpass et al [29], who proposed 3 types of researcher involvement in internet-based research: passive analysis, active analysis, and self-identified active analysis. Our study did not appraise methodological quality of risk of bias of the included articles since it is a scoping review.

### Stage 5: Collating, Summarizing, and Reporting the Results

We performed a descriptive analysis of the included studies. The analysis included data on publication date, country of origin, type of population, and type of disease studied.

## Results

### Search Results

**Figure 1:**
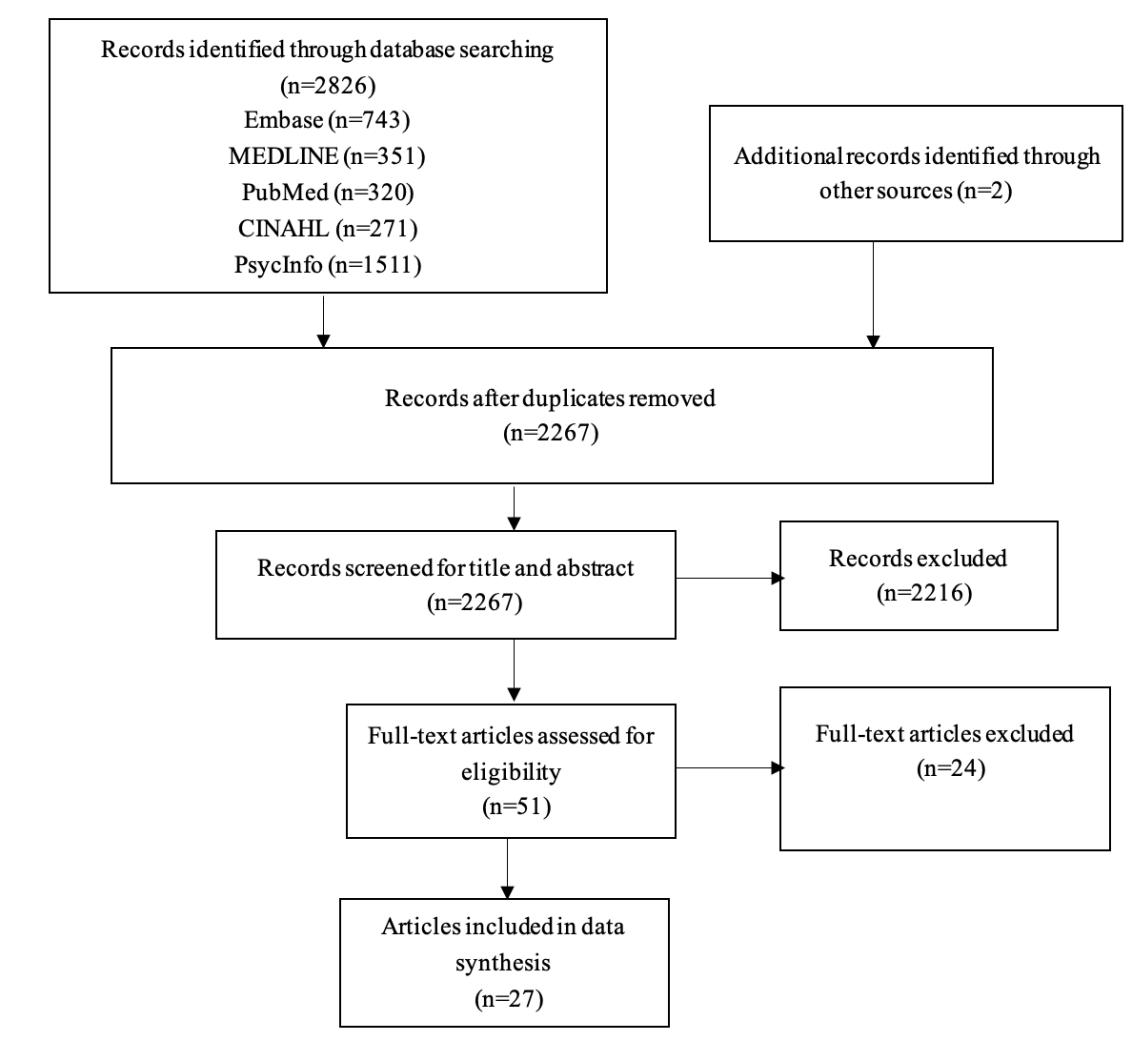
Flow diagram showing included studies.

The electronic database search yielded a total of 2836 records. The selection process was summarized in the PRISMA (Preferred Reporting Items for Systematic Reviews and Meta-Analyses) flow diagram as shown in Figure 1. Briefly, after removal of duplicates, the titles and abstracts of 2267 records were independently screened by 2 reviewers. The screening excluded 2216 irrelevant records. The full texts of the remaining 51 records were assessed for eligibility. After scanning the reference lists of 2 reviews, we evaluated the eligibility of 27 records whose full texts were screened. Out of these, 24 records were excluded for the following reasons: 9 did not involve people with chronic conditions, 10 were conference abstracts, 4 were not relevant human subjects, and 1 was a duplicate record. Finally, a total of 27 studies were included in the analyses (Multimedia Appendix 1)**.**

### Characteristics of the Included Studies

#### Publication Year and Geographic Distribution

Although our research evaluated studies from 2000 to 2022, none of the 27 included articles were conducted before 2009. As shown in Table 1, the number of publications grew through the years. This demonstrates that the application of online ethnography for people with chronic conditions has increased in the past 13 years. Most studies concentrated more on exploring the experience among the targeted population. Table 2 displays the distribution of articles by the continent of publication: 40.7% (11/27) of the articles [24,26,30-38] were from the United States, 29.6% (8/27) from England [25,39-44], 14.8% (4/27) from France [45-47], 7.4% (2/27) from Brazil [48,49], 3.7% (1/27) from Austria [50], and 3.7% (1/27) from Canada [51].

**Table 1:** The distribution of articles by the year of publication.

Year	Publications, n
2009	1
2011	1
2012	1
2013	1
2014	1
2016	3
2017	2
2018	1
2019	5
2020	5
2021	5
2022	1

**Table 2:** Distribution of publications by geographic location.

Geographic location	Publications, n
American	11
England	8
France	4
Brazil	2
Austria	1
Canada	1

#### Data Sources

Currently, most of the used data were from second-generation online social media, which include discussion forums, blogs, and social networking sites [52]. In our study, 4 online ethnographers collected data from more than 1 platform [25,26,38,41] (4/27, 14.8%), and most of them collected data from just 1 source. Among the retrieved articles, most data (10/27, 37.0%) were collected from online forums [33,34,40,43-47,50,53], 18.5% (5/27) were from Facebook [30], 11.1% (3/27) were from websites [30,39,54], 14.08% ((4/27) were from multisources, 7.4% (2/27) were from blogs [31,32], 3.7% (1/27) was from Instagram [37], 3.7% (1/27) was from Twitter [36], and 3.7% (1/27) was from Youtube [24].

#### Type of Researcher Involvement

Our analysis showed that the majority (24/27, 88.9%) of the included studies were passive analyses [24-26,30-34,36-38,40-44,46,48-51,53,54], 3.7% (1/27) were self-identified active analyses [45], and 7.4% (2/27) involved active analyses [35,39]. With regard to active analysis, Gibson et al [39] emphasized using participation-observation to complete data collection, and Frohlich [35] founded an online community and communicated with patients with inflammatory bowel disease. Troisoeufs et al [45] carried out 8 semidirective interviews from the consumers of online ethnography, and thus the study was identified as a self-identified analysis.

#### Domains of Chronic Conditions

On the basis of the report of the National Health Council [55], we classified the included chronic conditions into mental illness [24,41,44,51], female reproductive diseases [25,34,38,42], neurological disorders [40,45,46,50], cancer [31,32,39,53], rheumatic immune system diseases [33,37,43], common chronic diseases [26,36,48], autoimmune disorders [30,35], hereditary diseases [54], HIV [49], and thyroid disease [47]. Table 3 displays the distribution of articles by the domains of chronic conditions. Most of the studies focused on patients with mental illness (4/27, 14.8%), neurological disorders (4/27, 14.8%), cancer (4/27, 14.8%), or female reproductive diseases (4/27, 14.8%). Additionally, 11.1% (3/27) focused on diabetes, 11.1% (3/27) on rheumatic immune system diseases, 7.4% (2/27) on autoimmune disorders, 3.7% (1/27) on hereditary disease, 3.7% (1/27) on HIV, and 3.7% (1/27) on thyroid disease .

**Table 3:** The distribution of articles by type of disease.

Disease	Publications, n
Mental illness	4
Female reproductive disease	4
Neurological disorders	4
Cancer	4
Rheumatic immune system disease	3
Diabetes	3
Autoimmune disorders	2
Hereditary disease	1
HIV	1
Thyroid disease	1

#### Study Purpose and Important Results

We evaluated the effect of online ethnography on health information flow, emotional support, and interaction among patients with chronic conditions, peers, and health providers. We found the studies could be used to explore new modes of online medical practices, which include application of new medical technologies. With regard to study results, themes such as experience of self-management, experience of living with illness, and physician-patient relationship emerged (Multimedia Appendix 1).

#### Methods of Immersion and Data analysis

Our findings showed that the majority (17/27, 63.0%) of articles among the included studies did not report the immersion process in detail [25,30,33,34,37,38,40-42,44-46,49-51,53,54], while 37.0% (10/27) did report it [24,26,31,32,35,36,39,43,47,48]. With regard to data analysis, our results demonstrated that the studies employed various data analysis methods, including thematic analysis [31,33,38,40-44,47,48,53], grounded theory [24,35,38,50,54], content analysis [37,39,46,53], and disclose analysis [34]. The most used method was thematic analysis (11/27, 37.0%). Grounded theory (5/27, 18.5%) and content analysis (4/27, 14.8%) were also frequently used in the data analysis.

#### Ethical Issues

Ethical issues were mentioned in the majority of articles. Only 11.1% (3/27) of the articles did not mention any issues on ethics [26,45,50]. In general, there were 5 ethical issues in the included studies, which included public data [24,25,30-33,35-37,40,41,44,46,48,51,53], informed consent [38-40,42,43,47,49,51], privacy and confidentiality [31,32,40,41,44,46,47], naming [24,31,39-41,44,46,47,51,54], and legal consideration [24,25,30-34,36,37,39-44,47-49,54]. Moreover, 70.4% (19/27) of ethical concerns were approved or exempted by institutional review board, 55.6% (15/27) concerned public data, 37.0% (10/27) concerned naming, 25.9% (7/27) emphasized participants’ privacy and confidentiality, and 29.6% (8/27) acquired users’ informed consent.

## Discussion

### Principal Findings

Our findings demonstrated that online ethnography has great potential in the field of chronic disease research and has yielded beneficial outcomes in many chronic disease studies. This method has also attracted more attention from scholars in the field of chronic diseases**.** Most of the included articles in this scoping review were conducted from 2009 onward, and the number of included studies gradually increased with time, which may indicate growing interest in this area of research, especially since 2019. The included articles covered various chronic conditions, such as mental illness, neurological disorders, common chronic diseases, cancer, rheumatic immune system diseases, autoimmune disorders, hereditary diseases, female reproductive diseases, and HIV. These articles showed that online ethnography could highlight the patients’ experiences with disease and demonstrated new insights into improving medical practice from a patient-centered perspective. These diseases are characterized by long-term physical health conditions and the high mental health needs of the patients. In our scoping review, youth with mental illnesses and women with reproductive diseases were the most concerned population, which is consistent with Bour et al’s [56] study. These categories of patients are among the most stigmatized, marginalized, and vulnerable members of society and suffer from discrimination in many areas of their daily life. Online forums and social media can be a user-friendly environment where individuals can normalize their illness, assert their voice and identity with each other by validating shared experience with peers, and form social ties with a broad audience [57]. HIV patients and youth with mental illness affected by COVID-19 were also discussed in our study [44,49]. COVID-19 possibly shifts from an acute to chronic disease and also causes political controversy [58-60], while HIV has long been recognized as a health and political challenge [60,61]. Since online ethnography is currently a well-established method, which involves the social, cultural, and structural dimension of a given phenomenon [44], it is particularly well suited to dealing with personally or politically sensitive topics or illegal acts in online communities [19].

Online ethnography showed great contribution toward understanding how online users acquired health information, emotional support, and interaction with health providers, which provides thoughts on how to improve internet-based medical practices. First, the data showed that online users possibly sought health information and emotional support from peers instead of health care professionals. This could be because people tend to trust others who have similar challenges more than figures of authority from business, government, or mass media. However, there exists a controversy between the untrusted face-to-face patient-provider relationship and online support needs from health care professionals. Second, online ethnography can investigate the online health care pathways among chronically ill patients, which include individual and collective self-management and empowerment processes [53]. In addition, online ethnography can be used to evaluate social and experiential aspects of health technologies, such as deep brain stimulation and the Open Artificial Pancreas System (OpenAPS) do-it-yourself artificial pancreas technology [36,45,62], which could promote offline promotion of new medical technologies. In summary, online ethnography is a bridge that connects online and offline cultures, which affect offline-online social and medical practices among people with chronic conditions. It is also noteworthy that most of the studies were conducted in Western cultures. Therefore, there is need for research from other countries.

We also highlighted some characteristics and issues regarding the methodology and implementation process which should be dealt with. We identified online forums as the main data source, followed by Facebook, but the majority of the studies did not describe the immersion process. These data sources of online ethnography are broad and constantly evolving. Keim-Malpass et al [31] searched for blogs that explored the experience of women with cancer in the past, and now the use of online health forums for data collection for online ethnography is steadily increasing. This is because online forums can provide a rich source of primary naturalistic data about the perspectives and experiences of users with a particular health issue and can offer additional insights compared with traditional interviews [63,64]. Our analysis showed that Facebook has many users, and thus a large amount of data can potentially be harnessed. Although Facebook users’ activity consists of creating written posts, the potential use of text data for research is enormous, and thus online ethnographers are increasingly using it for research [49,65]. Instagram is pictorial, Reddit is text-based, and Twitter comprises short posts, all of which can facilitate sustained discussions where people share their in-depth experiences. Additionally, YouTube and video websites are public video-sharing platforms which may serve as important avenues to gain an understanding of people’s viewpoints [66-68]. Online ethnographers could choose one or more platforms as data sources depending on the research objectives. Moreover, using more than 1 research method was associated with superior outcomes compared to using a single study method. A combination of 2 methods can compare and explore deeper experiences and value perspectives [69]. For example, Lee et al [34] gathered narratives through interviews to circumvent the limitation of online ethnography where some women would keep silent or express false feelings in the infertility forum. The research methods for online ethnography are constantly evolving, and now it can stand alone or be combined with other research methods including offline interactions [70]. Furthermore, the majority of the studies did not describe the immersion process, a component that should be considered in future studies. Theoretically, the main researchers need to be immersed in the online culture of the targeted group prior to sampling. In our scoping review, only 8 articles described the immersion process; however, developing competencies as an online ethnographic fieldworker (immersive depth, prolonged engagement, researcher identification, and persistent conversations) is a key strategy for learning socially relevant events [71]. Reading relevant posts for several weeks or months, engaging researchers who are familiar with a given online environment, and being involved in the online community are 3 approaches to achieve a high quality of immersion. In addition, various qualitative data analysis methods were chosen based on specific research questions in the articles included, while quantitative methods need further exploration. Researchers believe that thematic analysis, lexical analysis, content analysis, grounded theory, narrative analysis, and “netnography” can be selected according to the research purpose [46,48]. In the future, there is a possibility that quantitative analysis could be combined with netnography [72]. Online ethnographers should take advantage of online software in data analysis under the condition that they follow the principles of ethnography.

We also highlighted 5 aspects of ethical issues and relevant limitations in the application processes of online ethnography for people with chronic conditions. First, the majority of our included studies were conducted using public data to avoid ethical controversy, which meant that individuals who had posted public messages did not have a reasonable expectation of privacy. However, such public data could lead to research bias, as passive patients with chronic conditions could have contrary opinions. Moreover, administrators of online communities sometimes would remove messages that were sad, shocking, or in violation of the group’s code of conduct, and, therefore, researchers could not capture a true and meaningful experience. Second, although it seemed justifiable to waive informed consent for observational research in a public space in the past [73], Hetland et al [74] recently suggested that informed consent was one way that ethnographers could handle potential risks in internet research. In our study, we discovered that some investigators had posted announcements or contacted users by email to protect users’ rights, which could be one form of balancing the issue of informed consent. Third, to protect the identities of a vulnerable population, all names were removed and post titles or specific demographic characteristics were not reported. We further recommend concealing discriminative descriptions because search engines are often capable of finding statements used in research reports, making it difficult to conceal certain data in certain avenues. Lastly, our finding showed that there was no consensus research among various national ethical committees regarding online ethnography [75]. Based on the literature, it was acknowledged that the ethics surrounding internet research is complex and multifactorial because internet research is a multidisciplinary project and has different types of environments [76,77]. Therefore, we suggest that online ethnographers should follow guidelines of local ethical committees. In conclusion, we recommend using public and private data as well as preserving patient anonymity, privacy, and confidentiality through removal of identified information when performing online ethnography among people with chronic conditions [78].

### Strengths and Limitations

The present study used a rigorous scoping review methodology based on the manual of the Joanna Briggs Institute. To ensure a broad search of the literature, the search strategy included 5 databases and employed the snowball technique. Although we highlighted significant findings, our scoping review process was limited by the fact that we might not have identified all relevant articles in the published literature despite attempts to be as comprehensive as possible. We also limited our review to documents written in English, which might have led to relevant studies being missed.

### Conclusions and Recommendations

Our finding suggested that online ethnography has good potential for exploring the deeper experience of people with chronic conditions, which is difficult to investigate with traditional ethnography. These results provide practical guidance for the online health care of chronic diseases in a wide range of fields. Researchers should first determine the research type and map the online community. There was high heterogeneity in the immersion, data collection, and analysis. We therefore recognize that online ethnography is adaptable and without strict methodological limitations. However, the analyzed articles demonstrated insufficiency in the immersion process into the online environment. In addition, we observed that there is no uniform standard for ethical concerns. Therefore, we recommend preserving the privacy and confidentiality of online users.

## Appendix

Multimedia Appendix 1 Characteristics of included studies.

## Abbreviations

OpenAPS: Open Artificial Pancreas System
PRISMA: Preferred Reporting Items for Systematic Reviews and Meta-Analyses
WHO: World Health Organization
