# Supplementary material for: Online Ethnography for People With Chronic Conditions: Scoping Review
Source: J Med Internet Res. 2022 Nov 29;24(11):e37941. doi: 10.2196/37941 (PMC9748796; doi:10.2196/37941)
Supplement: Multimedia Appendix 1 [file jmir_v24i11e37941_app1.docx]

**Table 1.** Characteristics of included studies

| **Author, year** | **Country** | **Study design** | **Target group** | **Domain of Chronic Condition** | **Type of researcher involvement^#1^** | **Data source** | **Methods of immersion** | **Data collection** | **Data analysis method** | **Study purpose** | **Important results** | **Ethic consideration^#2^** | **Description of limitations** |
| --- | --- | --- | --- | --- | --- | --- | --- | --- | --- | --- | --- | --- | --- |
| Copelton et al[22], 2009 | America | Online ethnography +  Interview | Patients with CD | Autoimmune disorders | Passive analysis | Discussion board websites | Unclear | First round:  294 discussion threads, January through July 2004  Second round:  Comparison | Inductive approach | To examine how laypersons give meaning to and act on symptoms, and challenge professional ways of knowing through the creation of lay standards of diagnosis. | ① Obtaining a diagnosis  ② Self-diagnosis  ③ Scientific self-diagnosis | ①⑤ | The findings of this ethnography methods could not be generalized beyond the samples employed |
| Gajaria et al[23], 2011 | Canada | Online ethnography | Youth with ADHD | Mental illness | Passive analysis | Facebook group | Unclear | 479 individually coded items | Constant comparison method | To examine what youth think about having ADHD by collecting data in a popular social networking site | ①the construction of group identify  ② creating an online support group  ③ defining the outgroup  ④ jokes about ADHD | ①④ | ①Researchers were unbale to ascertain actual age or gender of the individual who posted messages.  ②Researchers were able to ask follow-up questions to understand the statement made and pursue themes. |
| Keim-Malpass et al[24], 2012 | America | Online ethnography | Young cancer women | Cancer | Passive analysis | Blog | Lead author spent several months immersed in the online culture of young adults with cancer. | 16 blogs | Thematic analysis | To gain a unique perspective of the experiences of young women with cancer (age 20-39 years at diagnosis) through analysis of their online illness blogs. | Themes were identified as the women processed their diagnosis: living in the middle, new normal, urgency, and transition into the abyss | ①②③④⑤ | Results lack generalizability to a larger patient population. |
| Keim-Malpass et al[25], 2013 | America | Online ethnography | Young cancer women | Cancer | Passive analysis | Blog | Lead author spent several months immersed in the online culture | Snowball convenience sample, 16 blogs | Longitudinal narrative analysis | To investigate young women's experiences with complementary therapies through illness blogs | ① awakening  ② new identities (that  incorporate loss)  ③ the good stuff  ④ release. | ①③⑤ | ①Data lack generalizability beyond the experiences presented.  ②Identify was not captured. |
| Naslund et al[18], 2014 | America | Online ethnography | Individuals with severe mental illness | Mental illness | Passive analysis | YouTuBe | Reviewing relevant videos | 19 videos, 3044 comments | Grounded theory | To analyze how individuals with SMI use YouTube as a platform indicative of naturally occurring peer support. | ① minimizing a sense of isolation and providing hope;  ② finding support through peer exchange and reciprocity  ③ sharing strategies for coping with day-to-day challenges of severe mental illness  ④ learning from shared experiences of medication use ad seeking mental health care | ①④⑤ | Data trustworthiness |
| Gibson et al[26], 2016 | England | Online ethnography | Young people with cancer | Cancer | Active analysis | Video diary websites | Immersion +included 2 young people with previous cancer diagnosis for data collection | 18 young people, 156 films | Content analysis | To determine how young people describe challenges through a social media site | ① treatment and relenting side effects  ② rehabilitation and getting on with life  ③ relapse and facing more treatment  ④ coming to terms with dying | ②④⑤ | Not mentioned |
| Kirks et al[27], 2016 | England | Online ethnography | Young people with CF | Hereditary disease | Passive analysis | Charity websites | Unclear | 279 individuals,  151 discussion threads | Inductive grounded theory | To explore how online peer support is used by young people and parents to supports self-care in relation to CF | ①Managing treatments  ② Managing emotions  ③ Managing relationships  ④ Managing identity  ⑤ Managing support | ④⑤ | Background information about participants and their social context is largely absent, and responses could not be probed nor additional details elicited. |
| Willis et al[28], 2016 | America | Online ethnography +  Discourse analysis | Patients with arthritis | Rheumatic immune system disease | Passive analysis | Online forums | Unclear | 8231 posts | Inductive thematic analysis | To identify factors of self-  efficacy related to self-management behaviors found in CMC shared by people with arthritis in online health com-  munities. | ① Sharing disease experience  ② Suffering from disease symptom  ③ Asking for help | ①⑤ | ①This study cannot be generalized outsides of the four online health communities examined here.  ②This research examined only the CMC between community members and cannot predict the actual practice of self-management behaviors “offline” |
| Kendal et al[29], 2017 | England | Online ethnography | Young people with eating disorder | Neurological disorders | Passive active | Online forum | Unclear | 97 threads, 420 messages | Thematic analysis | To explore how young people used a youth-orientated, moderated, online, eating disorders discussion forum | ① Taking on the role of mentor  ② The online discussion forum as a safe space  ③ Friendship within the online forum  ④ Flexible help  ⑤ Peer support for recovery and relapse prevention | ①②③④⑤ | The dataset was from a 4-month period and any seasonal trends in the posts would not be identified from this study. |
| Lee et al[30], 2017 | America | Online ethnography +  Interview | Infertility patients | Female reproductive disease | Passive analysis | Online forum | Unclear | Three rounds | Discourse analysis | To examine how social support operates within this virtual realm, by examining how the forum’s language, norms, and values create and enforce categories of deserving  and belonging among site users | We find that the forum’s discourse privileges an  infertility narrative we term the ‘‘persistent patient,’’ in which a patient exhaustively  researches treatment options, undergoes multiple cycles of treatment despite repeated failures, and ultimately achieves success (a healthy baby) | ⑤ | The study was limited by potential selection bias in assessments of the types of patients present on the forum.  Users who were vocal in commenting on this forum were a self-selected set who are more like to have had failed cycles than is representative of the entire population of patients diagnosed with infertility. |
| Fernandes et al[31], 2018 | Brail | Online ethnography | Diabetes patients | Diabetes | Passive analysis | Facebook | Community mapping and two-week observation | 354 post， 1582 comments and 3326 likes | Thematic analysis | To understand how  online communities can contribute to increasing  the adherence of chronic patients to the treatment prescribed by the physician in Brazil. | ①Factors that affect adherence to treatment and what people with diabetes seek when participating in the online community  ② How can online health communities influence adherence to the treatment of chronic patients in Brazil | ①⑤⑦ | ①Data collection is restricted to what the participants choose to discuss and publicly disclose.  ②Researchers did not introduce themselves to community members during data collection can raise criticism about active participation in the virtual environment. |
| Frohlich et al, 2019[32] | America | Online ethnography | Patients with IBD | Autoimmune disorders | Active analysis | Facebook | Operating own online IBD community | 14 online IBD communities, interviewing 13 leaders. | Ground theory | To uncover how patient leaders of online IBD communities share health information, and how they conceive their responsibility to provide accurate information. | ①Criticism and Support of Medical Professionals  ②Sharing health information and medical advice  ③Community leaders’ responsibility for health information  ④Response from medical professionals to online communities |  | This study looked only at patient IBD communities, medial professional voices for IBD also exist online. |
| Litchman et al[33], 2019 | America | Online ethnography | Diabetes patients | Diabetes | Passive analysis | Twitter | Being involved and familiarized themselves with the OpenAPS community | 328 participants, 3347 tweets | Open-code approach | To understand how patients, caregivers, and care partners perceive OpenAPS, the personal and emotional ramifications of using OpenAPS, and the influence of OpenAPS on daily life in Twitter data. | ① OpenAPS use suggests self-reported AIC and glucose variability improvement  ② OpenAPS improves sense of diabetes burden and quality of life  ③ OpenAPS is perceived as safe  ④ patient/caregiver-provider interaction related to OpenAPS  ⑤ technology adaptation for user needs | ①⑤ | Self-report data may affect reliability |
| Nunes et al[34], 2019 | Austria | Online ethnography +  Observation+ Interview | Patients with Parkinson | Neurological disorder | Passive analysis | Online forum | Unclear | 332 posts | Grounded theory | To examine how patients and carers interact with their doctors in consultations and in an online community | ① Interactions between people living with Parkinson’s and their doctors (1) making explicit issues of concern (2) evaluating movement (3) discussing treatment adjustments (4) getting an understanding (5) getting inappropriate medication revised  ② Self-care technologies for interacting with doctors | ⑥ | Not mentioned |
| Tenderich et al[20], 2019 | America | Online ethnography | Diabetes patients | Diabetes | Passive analysis | Facebook, Twitter, YouTube, Instagram, Tumblr, Pinterest, Reddit, and Quora | Employing four researchers, including two college students who lived with T1D themselves. | Five rounds,  Roughly 450-500 social media content pieces | Unclear | ① To essentially “pull back the curtain” on what PWDs are doing on the social web by observing and analyzing in the broad landscape  ② To promote true understanding and empathy among those who work with diabetes  ③ To help provide a basis for the SM activities of organizations dealing with PWDs | Six vibrant themes regarding life with diabetes: (1) Humor, (2) Diabetic Pride, (3) Personal relationship with Diabetes Technology (4) Sharing Tips and Tricks  (5) Building Community  (6) Venting | ⑥ | The results of this netnographic study are not statistically representative of the full body of data. |
| Troisoeufs et al[35], 2019 | France | Online ethnography + Observation+ Interview | Patients with PD | Neurological disorder | Self-identified active analysis | Online forum | Unclear | Two rounds,  Unclear | Unclear | To describe the attention paid by users for medical information about DBS in the identity and self-help process | ① The three types of agents and roles posting on the Internet on the subject of Deep Brain Stimulation  ② Use of the information  (1) The search for information  (2) Mutual help and support  ③ Lived experiences and medical representations of DBS | ⑥ | Not mentioned |
| Cherif et al[36], 2020 | France | Online ethnography | Breast cancer patients | Cancer | Passive analysis | Online forum | Unclear | 967 reviews | Thematic and lexicomtric content analysis | To model the key stages underling the patient pathway and to identify the  challenging touch points of the interactions between patients and healthcare providers | Three stages: ①the discovery stage: emotional dimension regarding the patient and physician relationship ② examination stage: more technical and informational needs for the types of treatments  ③ Follow-up and survivorship stage: patient’s need to assess the treatments’ effectiveness and the quality of the follow-up | ① | Passive patients are not included in this study |
| Lavis et al[37], 2020 | England | Online ethnography +  Interview | Self-harm young people | Mental illness | Passive analysis | Twitter, Reddit, Instagram | Unclear | Two rounds, 10169 posts, 36934 | Iterative thematic analysis | To explore why a young person might engage with such content or about its impact on behaviour or well-being. | ① From offline to online: motivations for seeking self-harm content on social media  ② Online interactions: giving and receiving peer-support  ③ From online to offline: the value, impact and ambivalence of peer-support | ①③④⑤ | ①There is a need for ongoing research to keep apace as research papers will become out of data swiftly.  ②We did not examined cultural or geographical difference. |
| Lewis et al[38], 2020 | America | Online ethnography | Patients with RA | Rheumatic immune system disease | Passive analysis | Instagram | Unclear | 106 | Content analysis | To gain an understanding of Instagram use for image sharing related  to rheumatoid arthritis. | Social interaction and self-expression were the most frequently identified categories, suggesting that individuals use Instagram primarily for sharing awareness, sharing encouragement, and self-expression regarding rheumatoid arthritis. | ①⑤ | There could be a sampling bias related to the days selected. |
| Melo et al[39], 2020 | Brazil | Online ethnography | HIV patients | HIV | Passive analysis | Facebook | Unclear | 37 posts, 122 members | Unclear | To understand how the relationships between  chronicity and politics shape sociability and mutual help  among people living with HIV/AIDS. | ① Do the treatment and time will take care of the rest: mutual aid and HIV/AIDS as a chronic condition  ② Yes, there is danger around the corner, my dear: Politics, conflicts and sociality in the group | ②⑤ | Not having included here other data collection techniques such as semi-structured interview |
| Shah et al[40], 2020 | England | Online ethnography | Women with cerebral palsy | Female reproductive disease | Passive analysis | Facebook | Unclear | 45 members | Thematic analysis | To enhance understanding of the bodily and lifestyle effects of ageing with cerebral palsy (CP) for women, with a particular focus on experiences with sexual and reproductive healthcare (SRH) services in the UK and North America. | ① bodily effects of ageing  ② lifestyle effects of ageing  ③ experiences of reproductive and sexual healthcare | ②⑤ | The study may have been affected by self-selection bias  Participants’ posts could be seen by each other, potentially influencing other participants’ responses and potentially comprising anonymity. |
| Bayen et al[41], 2021 | France | Online ethnography | Patients with PD | Neurological disorders | Passive analysis | Online forum | Unclear | 70 users, 302 messages | Content analysis | To assess the users' characteristics, discussion contents, and the atmosphere of virtual peer communities. | ① Managing symptoms  ② Living with PD  ③Sharing illness experiences | ①③④ | ①The study population is not representative of the French general population  ②Due to the respect of users’ anonymity, their characteristic were difficult to describe, and our method does not allow to collect private posts, triggering a data loss |
| Fayn et al[42], 2021 | France | Online ethnography +  Interview | People with thyroid disease | Thyroid disease | Passive analysis | Online forums | 8 months of immersion | 12 people, 54 305 discussion threads | Thematic and lexical analysis | To examine the motivations that drive chronically ill  patients to engage in an individual and then collective empowerment process. | ① Individual empowerment: the first step towards collective empowerment  ② The power of the collective  ③ Community empowerment: building a network around shared identify  ④ Collaborative empowerment: skill building supported by actions  ⑤ Community empowerment: building a network around a shared identity | ②③④⑤ | This netnography only covered one forum and analyzed the activity of only 21 active patients |
| Fazekas et al[19], 2021 | England | Online ethnography | Patients with epilepsy | Female reproductive disease | Passive analysis | Twitter, Facebook, online forums, blogs | Unclear | 264 706 conversations | Qualitative in-depth and contextual analysis | To investigate the real-life experiences of PWE as reflected in online conversations on social media  and thereby raise awareness of the challenges and issues that are most important to PWE | ①disease awareness among the general public  ② the psychological and physical impact of seizures  ③ the importance of ensuing proper sleep  ④ understanding disease burden through time  ⑤ finding treatment and managing side effects  ⑥ dealing with depression and anxiety | ①⑤ | The study is also limited by not including other popular media platforms, and despite comprehensive research and piloting of the search syntax used, it is likely that additional key search terms were missed |
| Gonzalez et al[43], 2021 | America | Online ethnography | Women with pelvic organ prolapse | Female reproductive disease | Passive analysis | Twitter, Reddit, Instagram | Unclear | 3451 posts, 2088 users,  117 websites | Ground theory methodology +  Latent Dirichlet Allocation probabilistic topic modeling process | To characterize the decision-making process and illness experience of women with pelvic organ prolapse (POP) using large-scale social media analysis. | Allowing for information and support exchange and aiding in the decision-making process |  | ①The number of identified posts and generalizability could have been limited by the collection of websites  ②The quality of all websites from which the posts were identified is unknown. |
| Sloan et al[44], 2021 | England | Online ethnography | Patients with LUPUS | Rheumatic immune system disease | Passive analysis | Online forum | Seven members of the research team were fully immersed in the community over 30 years. | Forum posts form October 2019 | Thematic analysis | To give these patients a wider combined voice, and identifying unmet needs, values concerns and preferences, thus enabling patient-centered improvements to be considered. | (1) asking for advice on symptoms  (2) negative medical appointments/interaction  (3) medication/test results queries  (4) general advice given/sought  (5) emotional/mental health/struggling to cope | ②⑤ | The limitations are that the results may not be representative of the wider SLE population due to demographic and experience bias. |
| Winter et al[45],  2021 | England | Online ethnography +  Observation | Young people with mental illness during the period of COVID-19 | Mental illness | Passive analysis | Online forum | Unclear | 1033 posts,  13860 comments | Thematic analysis | To explore young people’s discussions on social media  about the impact of COVID-19 on their mental health in the UK. | ①The virus：loss, fear and other people  ②isolation and life inside the home  ③education at a distance  ④thinking about future | ①③④⑤ | ①There may be conversations in closed or private spaces which do not align with those that were analysed in this study   1. Demographics are not always reliable on social media. |

Note: #1: Active analysis refers to the researchers actively are involved in the participation and information exchange while passive analysis means researchers work as lurkers. Self-identify active analysis where researcher would further collect information through a more in-depth interview or clinic-based study.

#2: ①Public data; ② Informed consent; ③ Privacy and confidentiality; ④ Naming; ⑤ Legal consideration; ⑥ Not mentioned;
